# A Fresh Insight into Transmission of Schistosomiasis: A Misleading Tale of *Biomphalaria* in Lake Victoria

**DOI:** 10.1371/journal.pone.0026563

**Published:** 2011-10-24

**Authors:** Claire J. Standley, Christopher M. Wade, J. Russell Stothard

**Affiliations:** 1 School of Biology, University of Nottingham, Nottingham, United Kingdom; 2 Department of Zoology, Natural History Museum, London, United Kingdom; Royal Tropical Institute, Netherlands

## Abstract

Lake Victoria is a known hot-spot for *Schistosoma mansoni*, which utilises freshwater snails of the genus *Biomphalaria* as intermediate hosts. Different species of *Biomphalaria* are associated with varying parasite compatibility, affecting local transmission. It is thought that two species, *B. choanomphala* and *B. sudanica*, inhabit Lake Victoria; despite their biomedical importance, the taxonomy of these species has not been thoroughly examined. This study combined analysis of morphological and molecular variables; the results demonstrated that molecular groupings were not consistent with morphological divisions. Habitat significantly predicted morphotype, suggesting that the different Lake Victorian forms of *Biomphalaria* are ecophentoypes of one species. The nomenclature should be revised accordingly; the names *B. choanomphala choanomphala* and *B. c. sudanica* are proposed. From a public health perspective, these findings can be utilised by policy-makers for better understanding of exposure risk, resulting in more effective and efficient control initiatives.

## Introduction

Intestinal schistosomiasis is caused by the parasitic trematode *Schistosoma mansoni*, and is a neglected tropical disease of global importance [1]. The distribution and burden of the infection is determined to a large extent by the presence of the obligate intermediate hosts of the parasite, which are snails of the genus *Biomphalaria*. Although also present in Latin America and the Middle East, the vast public health burden of *S. mansoni* falls on Africa; within this context, Lake Victoria is a known regional hotspot.

Given their importance in determining the distribution and exposure risk of intestinal schistosomiasis, *Biomphalaria* snails (Basommatophora, Planorbidae) have long been the subject of intense scientific scrutiny. It was noted early on that different species of *Biomphalaria* appeared to transmit *S. mansoni* more or less successfully [2], [3]; of the 12 species of *Biomphalaria* in Africa, none are known to be completely resistant to infection, unlike some of the New World species [4], [5]. However, the African species have not been awarded the same level of research attention as the American *Biomphalaria* species, and there has long been confusion as to the status of various putative species and sub-species.

An attempt to rectify this taxonomic tangle was made in the late 1950s by Georg Mandahl-Barth, who used a combination of morphological characters to re-classify African *Biomphalaria*. Based on morphology, Mandahl-Barth proposed classifying the African *Biomphalaria* into four groups: the *B. pfeifferi* group, the *B. choanomphala* group, the *B. sudanica* group and the *B. alexandrina* group, each with numerous sub-‘species’ [6].

More recently, molecular tools have been used to try to elucidate the taxonomy of *Biomphalaria*. DeJong and colleagues [7] created a molecular phylogeny that completely contradicted Mandahl-Barth's four species groups, by showing that *B. choanomphala*, *B. sudanica* and *B. alexandrina* were actually more closely related to each other than to the other species in their respective ‘species groups’ as defined by Mandahl-Barth. This cluster of *B. sudanica*, *B. choanomphala*, *B. alexandrina* and *B. smithi* was termed the ‘Nilotic species complex’; more recent molecular work on East African *Biomphalaria* has supported this clade, although some inconsistencies have been observed when also considering morphology [8], [9].

In Ugandan Lake Victoria, snails identified, based on morphology, as *B. choanomphala* are considered the main transmitter of the parasite, as no *B. sudanica*-like snails have ever been found naturally infected in that portion of the lake. Indeed, early laboratory studies suggested they might be refractory to infection with *S. mansoni* [10]. However, transmission by *B. sudanica* is common in the Kenyan and, to a lesser extent, Tanzanian portions of Lake Victoria [11]–[13], although much of this evidence is anecdotal rather than formally observed; many studies on *in situ* transmission do not attempt robust identification of the snails observed. Moreover, the relationship between the two species as found in the lake has never been investigated in detail, and certainly not since the advent of molecular tools.

The desire to identify accurately the species of *Biomphalaria* in Lake Victoria is linked directly to the need for greater understanding of the transmission patterns of *S. mansoni* in the lake. Intestinal schistosomiasis is rife in communities living by the lakeshore [14]–[16] and by understanding the dynamics of the snails responsible for transmission it will be possible to identify possible exposure hot-spots, and direct treatment and education interventions to these areas.

## Results

### Survey sites and snail collection

**Figure 1:**
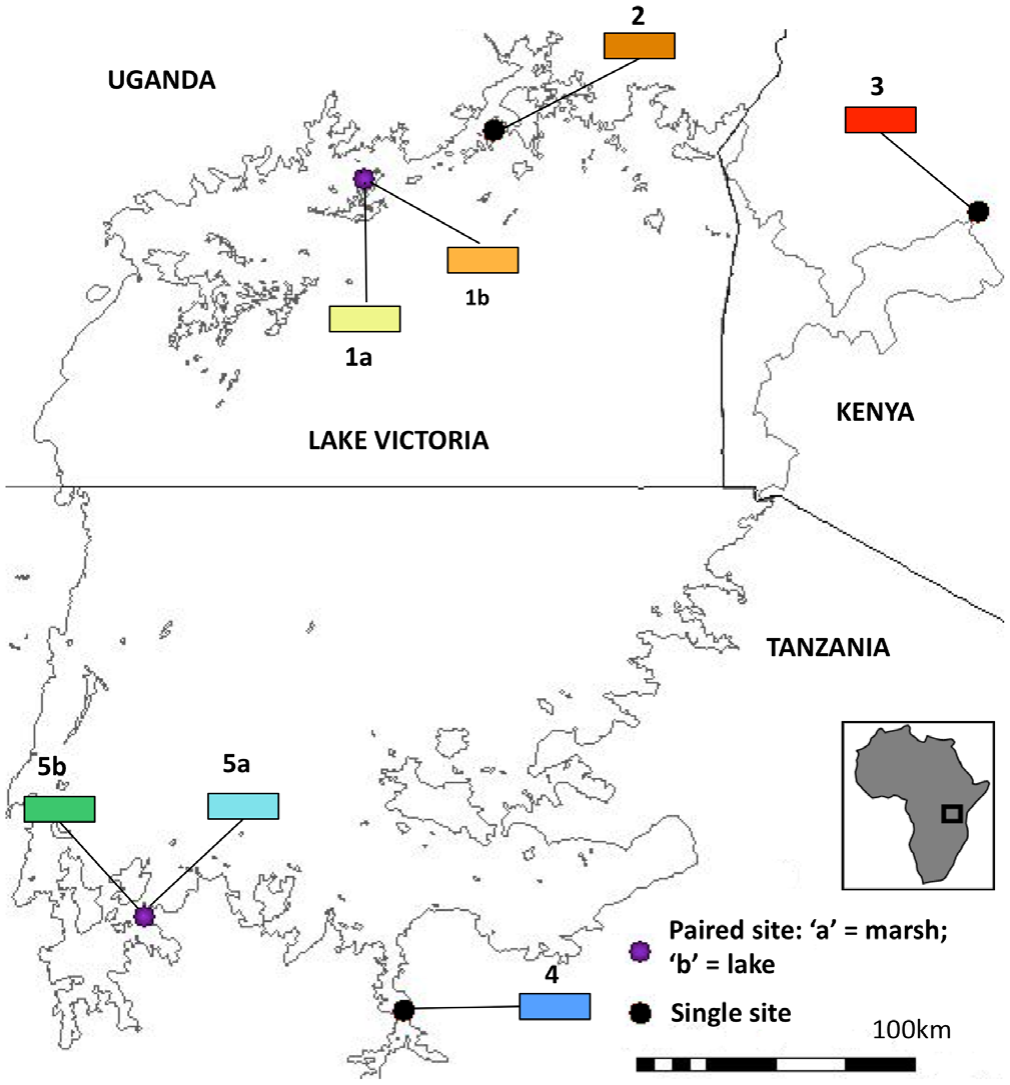
Map of the sites included in the taxonomic and phylogenetic analyses. The ‘paired’ sites are indicated by a purple circle whereas single sites are marked by a red circle. Paired sites consisted of a marsh habitat separated from the lake proper, whereas single sites only had one habitat that was sampled.

Snails were collected from seven sites along the shoreline of Lake Victoria; three in Uganda, three in Tanzania and one in Kenya (Figure 1). Four of the sites were ‘paired’, meaning that a marsh site and a lake site were located adjacent to each other, at virtually the same geographical coordinates. Field identifications of snails were based on morphology: *B. sudanica-*like snails were observed mostly at marsh sites whereas *B. choanomphala*-like snails were collected almost exclusively from the lake proper (Table 1).

**Table 1:** –Table of geographical locations used for taxonomic analysis.

Site	GPS coordinates	Habitat	*Biomphalaria* morphotype observed
**1a**	N0.01477;E32.76717	Lake	*B. choanomphala-*type and *B. sudanica-type*
**1b**	N0.01477;E32.76717	Marsh	*B. choanomphala-*type and *B. sudanica-type*
**2**	N0.17320;E33.18397	Lake	*B. choanomphala*-type
**3**	S0.09589;E34.74907	Marsh	*B. sudanica*-type
**4**	S2.713467;E32.89392	Marsh	*B. sudanica*-type
**5a**	S2.40525;E32.05935	Marsh	*B. sudanica*-type
**5b**	S2.40525;E32.05935	Lake	*B. choanomphala*-type

### Molecular results

No significant variation was seen in ITS banding pattern between Lake Victorian individuals, although the restriction enzyme digest of this nuclear DNA region could differentiate between these individuals and Argentinean *B. peregrina*, included as controls. Overall, the COI and 16S data produced the same patterns; as such, only the COI results will be displayed in full here (data for 16S can be found in the Supplemental Information). Of the 655 base pairs of the COI gene used in the analysis, 135 positions were variable and 109 were parsimony-informative. 36 unique COI haplotypes were recovered from 75 samples. All haplotypes were compared to the existing database of haplotypes as calculated for a larger set of Lake Victoria *Biomphalaria* sequences (GenBank accession numbers HM769132.1-HM769258.1) and matched accordingly.

For the COI sequences, the mean corrected distance across the dataset as a whole was 2.0%. The within-morphogroup means were very similar to the between-morphogroup mean distances (Table 2; see also Table S1 for 16S gene corrected distances). In all cases, the GTR+G model of nucleotide substitution was used, based on the model testing as described in the Methods section.

**Table 2:** – Table of COI corrected mean distances within samples grouped by morphogroup.

Morphogroup	*B. choanomphala*-like	*B. choanomphala*-intermediate	*B. sudanica*-like	*B. sudanica*-intermediate
***B. choanomphala*** **-like**	*0.020*			
***B. choanomphala*** **-intermediate**	0.023	*0.022*		
***B. sudanica*** **-like**	0.023	0.026	*0.020*	
***B. sudanica*** **-intermediate**	0.018	0.021	0.018	*0.015*

The diagonal (in italics) shows the within-morphogroup mean genetic distance. The model of nucleotide substitution used for the correction was GTR+G.

The average genetic distance within the samples was comparable to that within other *Biomphalaria* species. For COI, there was 1.6% difference across the Lake Victorian samples, as compared to 1.8% between African-wide samples of *B. pfeifferi* and 3.4% difference between three sequences of *B. glabrata*, from South America as well as laboratory strains (Genbank sequences DQ084823.1, DQ084866.1 and DQ084824.1). Distances between the Lake Victorian samples and other species were also comparable to inter-specific distances between other *Biomphalaria* species. Difference compared to *B. alexandrina* was lowest, at 1.7%, but otherwise for the other African *Biomphalaria* it ranged from 2.9% to 3.7%. South American species, excluding *B. glabrata*, were between 4.8% and 6.8% different.

**Figure 2:**
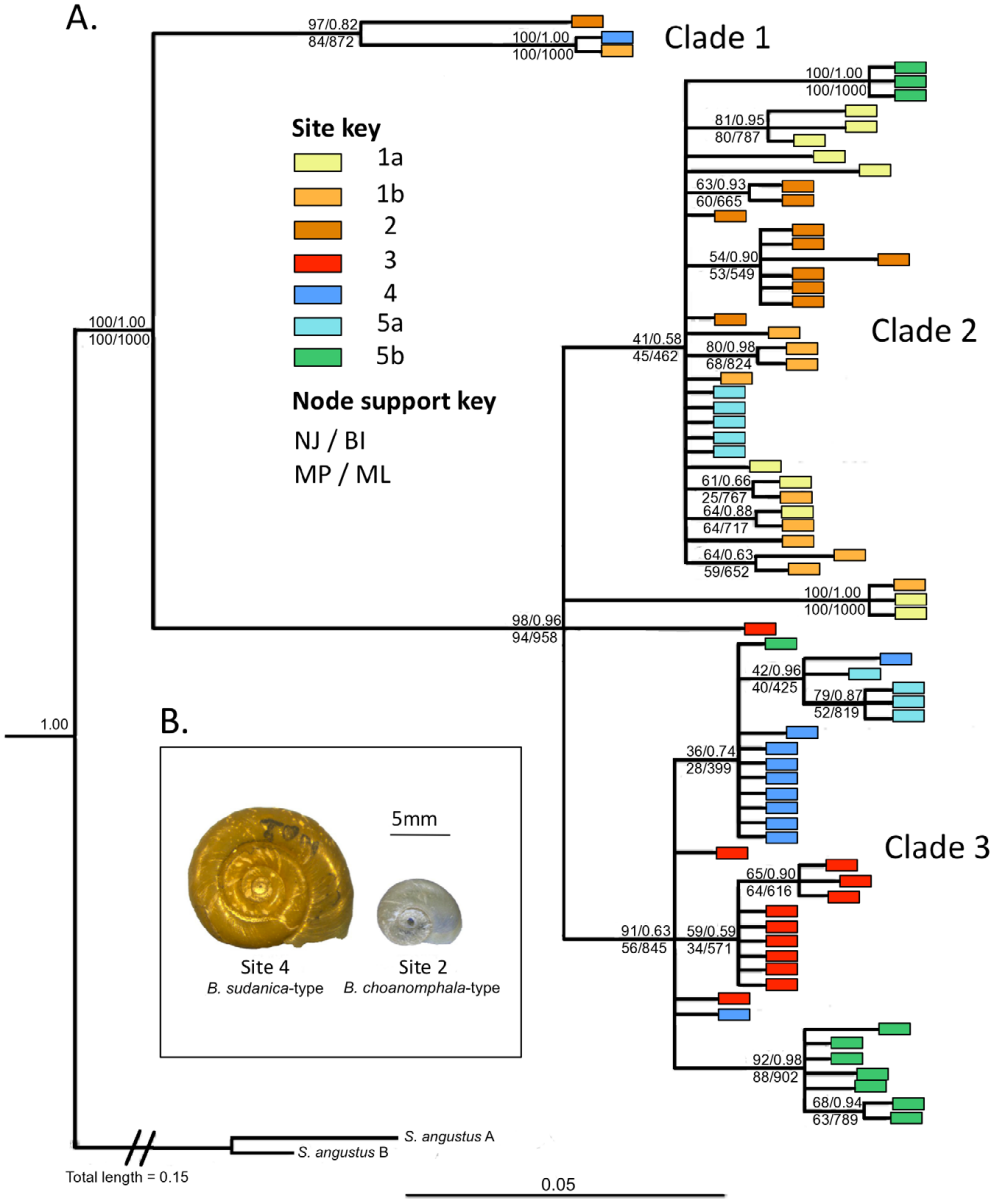
Phylogenetic tree of COI sequence data. The branch lengths and general topology are based on the Bayesian inference tree; node support values are those that were >50% based on the Bayesian posterior probabilities, and neighbour-joining, maximum likelihood and maximum parsimony bootstrap support values have also been added, as per the legend. Two sequences of *Segmentorbis angustus*, also from Lake Victoria, were used as outgroups. A. (inset) Example of the overlap of morphotypes within genetic clades on the tree. Both of these snails formed part of the highly divergent clade 1 on the tree, and yet are typical examples of a *B. sudanica* morphotype (left) and *B. choanomphala* morphotype (right).

The phylogenetic tree showed genetic differentiation of certain groups, but not conforming to the putative, morphologically-based, species identifications from the field. In the COI tree, the samples are split into three main clades, with *B. choanomphala*-like and *B. sudanica*-like morphotypes in all groups. Some of the more derived branching, towards the end of the clades, separated samples into their respective sites (Figure 2A; see also Figure S1 for equivalent analysis of 16S gene).

The example of cross-over of morphotypes within clades is clearly shown when looking at clade 1, which is well-supported and strongly differentiated. Three indivduals from sites 1b, 2 and 4 form this clade; in spite of their close relatedness and high divergence from the other samples, they comprise of two *B. sudanica*-like snails (site 1b and 4) and one typical *B. choanomphala*-like individual from site 2 (Figure 2B [insert]).

When the constrained neighbour-joining trees were created for hypothesis testing of monophyly, the trees with enforced monophyly for either *B. choanomphala*-type and *B. sudanica*-type snails were significantly less likely than the unconstrained trees (p<0.001, for both constraints). These results statistically support the presence of mixed morphotype clades that had been visually identified on the trees.

**Figure 3:**
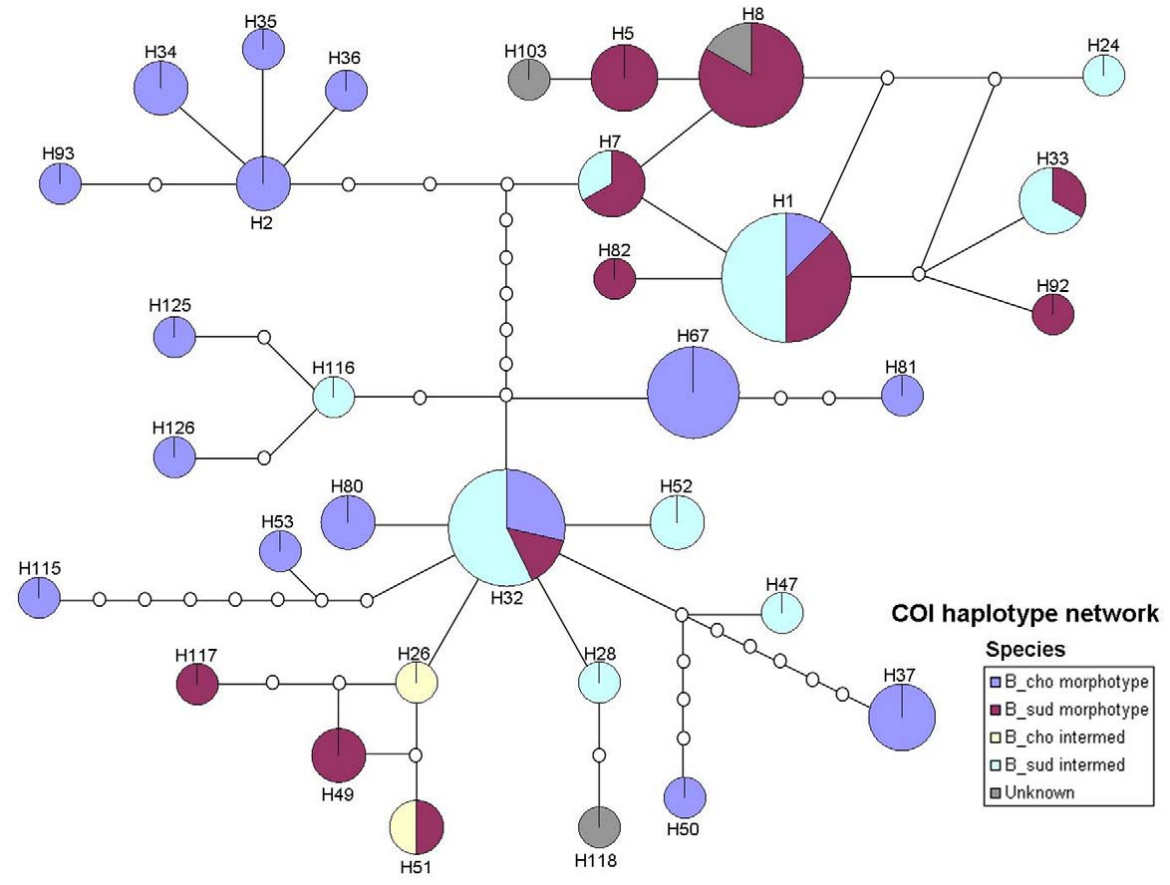
TCS Minimum spanning network of the 37 unique COI haplotypes. The size of the circle is proportional to the number of sequences which matched that haplotype; the colours of the pie slices indicate the proportion of each morphotype among the total snails of that haplotype.

The COI network showed two main groups, with one clustering around H1 and H8 and the other around H32 (Figure 3; see Figure S2 for data from 16S gene). However, both groups have snails of *B. choanomphala* and *B. sudanica* morphotypes. In fact, in several cases, unique haplotypes were even shared by different morphotypes. The network also shows that abundant haplotypes tend to be central nodes with less common haplotypes radiating out from these abundant forms.

### Morphological results and PCA

**Figure 4:**
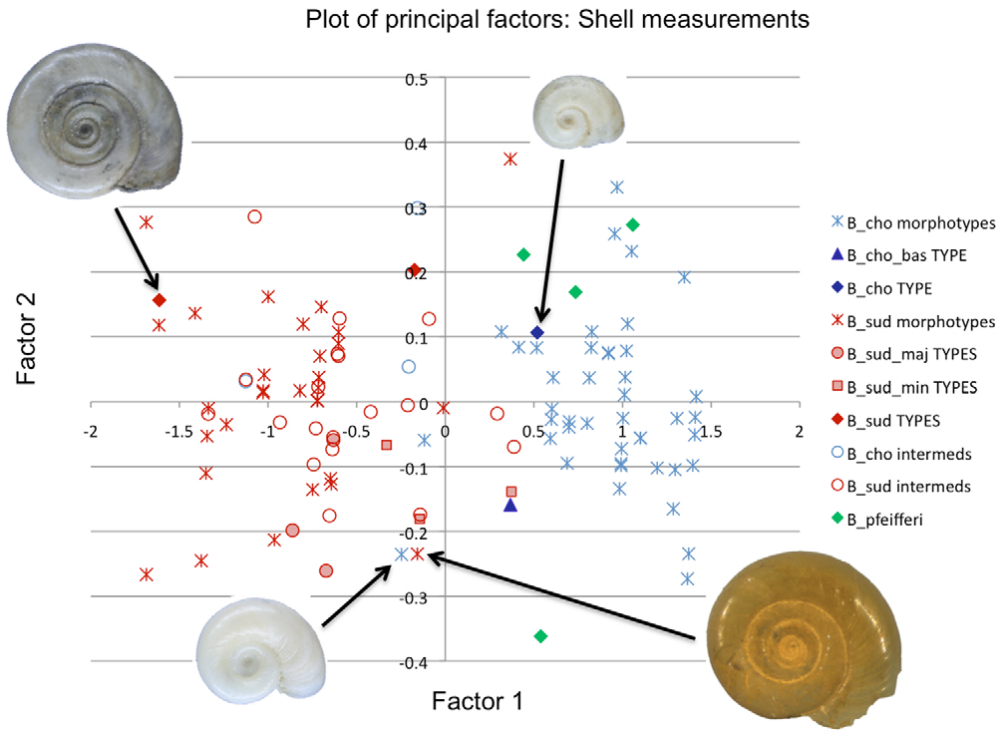
PCA plot showing the first two eigenvalues (‘Factor 1’ and ‘Factor 2’) for the shell measurement analysis. These two factors explained 99.8% of the variation in the data. The shell pictures demonstrate the visual form of the shell corresponding to that particular coordinate on the plot. The various different morpho-groups (Lake Victoria samples versus ‘type’ material from Berlin versus field-caught B. pfeifferi) are indicated as per the key. The abbreviations for the species names are as follows: ‘B_cho’  =  B. choanomphala; ‘B_cho_bas’  =  B. choanomphala var. basinulacatus ‘B_sud’  =  B. sudanica; ‘B_sud_maj’  =  B. sudanica var. major; ‘B_sud_min  =  B. sudanica var. minor; ‘intermeds’  =  intermediate forms.

The PCA of the two conchological datasets showed rough divisions between the *B. choanomphala*-types and the *B. sudanica*-types, with the *B. pfeifferi* samples clustered within the *B. choanomphala* morphospace (in the interest of space, Figure 4 presents the shell measurement data, which was the more complete dataset. The aperture data PCA is included as Figure S3). The ‘type’ material from the Museum für Naturkunde, in Berlin, clustered centrally within the rough groupings of morphospecies, apart from one *B. sudanica* var. *minor* type specimen, which was quite distant from the rest of the *B. sudanica*-like samples and moreover was very close to the *B. choanomphala* var. *basinulacatus* type specimen. There was cross-over between the two main regions of morphospace, particularly with intermediate forms of both morphotypes but also with a few specimens which had been identified as morphologically “true”.

Note the proximity of morphospace shared by the bottom two shell pictures; although grouped closely together, visually the shells appear very different and clearly characteristic of a *B. choanomphala*-like (on the left) and a *B. sudanica*-like snail (on the right).

The internal morphology measurements did not reveal any clear groupings and few conclusions could be drawn from these data (Figure S4).

### ANOVA and matrix correlations

The ANOVA tests and associated Tukey HSD supported the PCA findings of an overlap between morphotypes. They also strongly associated particular morphological features with certain habitat types. The significance tests between the means of the shell measurements for the various species groups showed that the *B. choanomphala* and *B. pfeifferi* have quite similar ratios between their dimensions, except for the depth ratio, in which they are significantly different (Table 3).

**Table 3:** – Significant results for Tukey HSD tests on shell measurements.

Ratio	Comparison	Difference	Lower CI	Upper CI	adjusted p-value
Height/Width	cho/sud	0.049	0.031	0.068	0.000
	cho/cho int	0.071	0.022	0.121	0.001
	cho/sud int	0.047	0.024	0.070	0.000
	sud/pfei	−0.059	−0.103	−0.015	0.003
	cho int/pfei	−0.081	−0.144	−0.017	0.006
	sud int/pfei	−0.056	−0.102	−0.011	0.008
	lake/marsh	0.032	0.014	0.050	0.000
	stream/marsh	−0.068	−0.119	−0.016	0.007
Depth 1/Depth 2	cho/sud	0.110	0.081	0.139	0.000
	cho/sud int	0.109	0.073	0.145	0.000
	cho/pfei	0.082	0.013	0.151	0.012
	lake/marsh	0.058	0.025	0.091	0.000
Depth 2/Width	cho/sud	−0.133	−0.160	−0.106	0.000
	cho/cho int	−0.102	−0.174	−0.029	0.002
	cho/sud int	−0.117	−0.150	−0.083	0.000
	sud/pfei	0.125	0.060	0.189	0.000
	sud int/pfei	0.108	0.041	0.175	0.000
	lake/marsh	−0.069	−0.103	−0.034	0.000
	stream/marsh	0.112	0.015	0.209	0.020

‘cho’ stands for *B. choanomphala-*type snails, ‘cho int’ stands for *B. choanomphala-*intermediates, ‘sud’ stands for *B. sudanica*-type snails, ‘sud int’ stands for *B. sudanica-*intermediate, and ‘pfei’ stands for *B. pfeifferi* snails.

*B. pfeifferi* is also significantly different from all the other morphogroups for the height/width ratio, and from both *B. sudanica* types (the ‘true’ form and the intermediate forms) in terms of depth 2/width ratio. Otherwise, it is the ‘true’ *B. choanomphala*-types that were most often significantly different from the other morphotypes. *B. choanomphala* intermediates were not significantly different from the *B. sudanica* intermediates for any of the measurement ratios, and nor were ‘true’ *B. sudanica* and the *B. sudanica* intermediates. The most striking result came in terms of testing for significance based on habitat: all three ratios were significantly different between snails from the lake habitats versus those from the marsh habitat. The stream habitat was also significantly different from the marsh habitat, when testing the height/width and the depth 2/width ratio.

The matrix correlation tests between the average pair-wise distances of the morphological measurements and the two different genetic markers were not significant. This means that the groupings seen in the genetic data cannot be significantly correlated to differences in the morphological characters that were analysed, or vice versa.

## Discussion

The combination of the molecular and morphological data suggests that the accepted view of *B. choanomphala* and *B. sudanica* as separate species in Lake Victoria needs to be revised, while the significance of habitat points to a strong environmental influence on phenotype and a reconsideration of the potential zones of transmission along the lakeshore.

The high levels of variation in both the mitochondrial genes tested were in marked contrast to the negligible variation seen in the ITS region. ITS digests have been used successfully for species identification with South American *Biomphalaria* [17], [18]; in this case, the almost uniform nature of the banding patterns for the ITS digest lends weight to the hypothesis that the samples do not represent two distinct species, which was also supported by the genetic distance data.

The morphological measurements revealed some evidence of groupings that were consistent with the putative field identification as either a *B. sudanica*-or *B. choanomphala*-type snail, although the small number of measurements used should be taken into consideration. Even so, there were also overlaps in morphospace between the two groups, even from type specimens, as in the case of *B. choanomphala* var. *basinulacatus* in the shell measurements and aperture outline PCAs. Overlap of *B. pfeifferi* in morphospace with other *Biomphalaria* types has been reported previously [8], but generally *B. sudanica*-type shells are considered easy to distinguish from the rapidly-whorling forms. However, the overlap seen in the shell measurement and aperture outline PCAs between both *B. sudanica*-types and *B. choanomphala* types suggest that statistically the two forms are difficult to differentiate, and in fact intermediate forms may be found on a continuum between the extremes exemplified by the type specimens.

A very interesting finding was that many of the differences in the average measurements were significant between habitat types. Although causation is far from certain, this evidence could tentatively suggest that habitat might be a factor in driving the morphological plasticity seen in the dataset. Shell shape is known to be strongly environmentally determined in many snail species [19]–[22] so it would not be difficult to imagine factors associated with habitat driving ecophenotypic variation in *Biomphalaria* in Lake Victoria.

Here, the majority of *B. sudanica*-type snails were found in marsh-like habitats, whereas *B. choanomphala*-type snails were located in the lake proper. Of course, many ‘good’ species are confined to a particular habitat type, and so this finding is not that surprising; however, the genetic evidence showing complete cross-over between these morphotypes suggests that the *Biomphalaria* from Lake Victoria should be considered one, highly diverse species, both in molecular and morphological terms. The ‘species’ as they appear from putative field identifications could in fact be adaptations, based on some inherent plasticity in morphology, to environmental cues. It has been hypothesized that this has occurred in Lake Albert, as well as elsewhere in Uganda, where snails putatively identified as *B. pfeifferi*, based on shell characters, were in fact molecularly part of the Nilotic species complex [9]; similarly a snail with a typically *B. stanleyi*–like morphology from Lake Albert had a DNA sequence that clustered closely with *B. pfeifferi* [8]. In this latter case, it was suggested that the rapidly increasing whorls of the *B. stanleyi* morphotype could be an adaptation to lacustrine conditions; *B. choanomphala*-type snails have a very similar form and as such the same process could be occurring in Lake Victoria.

The high levels of genetic distance observed between *B. sudanica* elsewhere in Africa as compared with the Lake Victorian samples analysed here has implications for the revision of the nomenclature for these *Biomphalaria. B. sudanica* was actually described in 1870 by von Martens, 9 years before he also described *B. choanomphala*. As such, based on the rules applied by the International Commission for Zoological Nomenclature (ICZN; www.iczn.org), ‘*B. sudanica’* should take precedence over ‘*B. choanomphala’*. However, given that *B. sudanica* elsewhere is likely a true species, based on the genetic distance observed between the Lake Victoria samples and those on Genbank, whereas *B. choanomphala* is considered endemic to Lake Victoria, we propose that ‘*B. choanomphala*’ should take priority. The two different morphotypes, as identified by the main morphological groupings observed by the shell measurement and aperture analyses, should be denoted as variants. As such, *B. choanomphala-*type snails, and close intermediate forms, should be referred to as *B. choanomphala choanomphala* whereas *B. sudanica* and its intermediate forms should be described as *B. c. sudanica*.

If considered one species, then despite evidence to the contrary from some parts of Lake Victoria [11], [23], both variants of *Biomphalaria* should be considered equally capable of transmitting *S. mansoni*, until more detailed population-level compatibility experiments prove otherwise. Indeed, informal compatibility experiments were conducted in the field on some of these populations, whereby a local isolate of *S. mansoni* miracidia were used to infect snails from sites 5a and 5b (see Table 1 and Figure 1). Snails from both sites proved to be compatible with the parasite; after 19–25 days, individuals from both site 5a and site 5b started shedding cercariae, which were able to infect laboratory mice, thus proving their viability. However, we believe that until this is shown more completely, it remains important to distinguish between the two morphotypes through their nomenclature, for purposes of experimental design.

If *B. sudanica* and *B. choanomphala* are two forms of the same species and both equally able to transmit *S. mansoni*, then there are clear implications for the control of intestinal schistosomiasis in humans. To date, many national control programmes have included educational messages alongside mass chemotherapy, in order to change water contact behaviours and reduce exposure to the disease [24], [25]. On the whole, the message has focused on reduction of contact with freshly-collected lake water, for example through standing it for 24 hours prior to use, or, as a more long-term strategy, by installing water bores and pumps. However, if *B. sudanica*-type snails are also capable of transmitting, then the habitats that these forms frequent should be considered a high-risk zone for transmission. This includes marsh areas set back from the lake, which are often crossed while going to and from the lake, and where livestock are often grazed. Adding information about the risks of these marsh areas would assist in controlling exposure risk in this ‘new’ transmission environment. Similarly, in some cases it may be possible and desirable to drain marsh areas, given that they can also be important habitats for mosquito larval development, and thus implicated in the transmission of other diseases, such as dengue fever and malaria. Moves such as this would be an immediate, and highly beneficial, public health effect of this research.

## Materials and Methods

Molecular data from two mitochondrial markers (COI and 16S) and one nuclear region (ITS) were amplified from snails from seven sites around Lake Victoria. The snails were specifically preserved so that morphological analyses could also be undertaken on those same individuals, for a detailed insight into the connection between the genotype and phenotype of the samples.

### Sample collection and site selection

7 locations were chosen for inclusion in the taxonomic study on the basis of geography, habitat, abundance of snails and putative field-identified species. Species identifications in the field were made through shell morphology, based on two published keys [4], [26]. Three of the sites chosen contained *B. sudanica*-type snails, two contained *B. choanomphala*-like snails and two a mixed group of both. Snails collected from these seevn sites were putatively identified as ‘true’ *B. choanomphala*-types, ‘intermediate’ *B. choanomphala-*types, ‘true’ *B. sudanica*-types or ‘intermediate’ *B. sudanica*-types. Please see Figure 1 and Table 1 in the Results section for details of the geographic location of the sites as well as the types of snails found in each and the habitat type.

The snails were then shed to examine for infection with parasites before being relaxed overnight in mentholated water, killed and dissected to preserve the whole shell, a portion of tissue in alcohol and the bulk of the body in Raillet’s solution. 10–12 individuals per site were included both the molecular and morphological analyses.

### Molecular methods

Genomic DNA was extracted from between 10 and 12 snails from each of the designated 7 sites, using a standard CTAB protocol [27]. Separate PCRs were performed to amplify fragments of the cytochrome oxidase sub-unit 1 (COI) gene, the 16S ribosomal RNA gene (16S) and the whole internal transcribed spacer region of the nuclear genome, using standard universal primers (LCO1490 and HCO2198, 16arm/16brm and ETTS1/ETTS2) and cycling conditions [28]. Amplifications were done using Promega Go-Taq (Promega Corporation, Madison, UK) in a 25 µl reaction. All successful amplifications were purified using a Millipore PCR_96_ Cleanup kits on a vacuum manifold (Millipore, Billerica, USA) as per manufacturer's instructions, using pure water for washing and resuspension. Product concentration was quantified on a Nanodrop ND-1000 Spectrophotometer (Nanodrop Technologies Inc., Willington, USA), and sequencing reactions were performed on mitochondrial purified PCR products using an Applied Biosystems Big Dye Kit (version 1.1) and run on an Applied Biosystems 3730 DNA Analyzer (Applied Biosystems, Carlsbad, USA). Sequences were assembled and edited by eye using Sequencher v 4.8 (Gene Codes Corporation, Ann Arbor, Michigan, USA: http://www.genecodes.com).

The ITS fragment was also sequenced for a few individuals (specifically, the ITS1 segment was sequenced), but was not very successful and showed potential for having multiple copies in a single individual; in this instance it is difficult to determine whether sequences between individuals are homologous and can be compared. As such, instead of direct sequencing, the entire amplified ITS fragment from all the samples was used in a random fragment length polymorphism restriction enzyme digest, to look for point mutations this way. 2.4 µl of unpurified PCR product was added to a mixture containing 2 µl (2 units) of the restriction enzyme (*Alu*I or *Hae*III), 2.4 µl of buffer solution appropriate to that enzyme and 17.6 µl pure water. The mixture was left to digest overnight at 37 °C, then 15 µl of it was run on a 4% agarose gel stained with GelRed^TM^.

The mean corrected genetic distances were calculated for within and between each of the morphogroups. The best-fit model of nucleotide substitution for the data was determined by estimating the likelihood of neighbour-joining distance trees built using various models (HKY, HKY+G, GTR and GTR+G) then comparing these sequentially using a Chi-squared test. The genetic distance across the whole dataset was also evaluated and compared to that within other *Biomphalaria* species, from sequences retrieved from GenBank; mean distances were also calculated between the Lake Victoria dataset and other species, again to ascertain whether levels of genetic distance seen were comparable to intra- or interspecific variation.

Four separate methods for determining relationships between taxa were used to build branching trees of the mitochondrial data: distance (neighbour-joining), maximum likelihood, maximum parsimony and Bayesian inference. The last 10% of trees were used for calculation of posterior probabilities on the Bayesian inference tree and node support was calculated by bootstrapping using the other three methods (1000 replicates for each).

In addition, two more neighbour-joining distance trees were created for each mitochondrial gene. These were used to see whether the resultant tree was more or less likely if monophyly of the morphogroups was enforced. The first tree was built with a constraint that all specimens which had been putatively identified as *B. choanomphala*-like be put together in a monophyletic group; the second one's constraint was that all *B. sudanica*-like snails would be monophyletic. The likelihood values of these trees could then be statistically compared to that of the unconstrained COI and 16S trees, to see if there was statistical evidence of either morphotype being a monophyletic grouping.

The alignments of sequences for both COI and 16S were used to create two minimum spanning distance networks, with a connection limit set to 95%. The abundance of each unique haplotype and the proportion of morphotypes (based on putative field identification) at each node was noted and included in the final network for each gene.

### Morphological methods

Three separate sets of morphological measurements were taken to compare with the genetic findings. These included conchological measurements, aperture outline analysis and internal morphological measurements. All length measurements were log-transformed prior to further analysis.

Photos of each shell were taken using a camera attachment to a light microscope and the number of whorls counted on-screen, at high magnification. Shell height, width, depth below the umbilicus (hereafter called ‘depth 1’) and depth at the highest point (‘depth 2’) were all measured using digital calipers, accurate to 0.01 mm. The outline of each aperture was marked out with 50 equidistant points were converted into phi functions to describe the shape of the curve. Using specimens that had been removed from their shells in the field and their bodies placed in Raillet's solution, further dissections could be made in order to compare internal anatomy. Samples were dissected under a light microscope to reveal the copulatory structures, and in particular, the penis sheath/preputium and the prostate gland. The lengths of the penis sheath and the preputium were noted using a camera lucida microscope attachment, and the prostate dissected further to count the number of diverticula.

In addition to the field collected snails from the sites described in Figure 1, a number of type specimens from the Berlin Museum für Naturkunde were also measured and included in the analysis. These included the single “type” specimens for each species, as well as any holotypes or syntypes that were available (designated “subtypes” in the text). For comparative purposes, field-collected *B. pfeifferi* (from Zambia) and *B. sudanica* from Lake Albert were also included as putative outgroups.

### Principal component analysis, ANOVA and matrix correlations

Principal component analysis (PCA) was performed on all three morphological datasets in R [29]. ANOVAs were run to test directly for significance between various measurements and the putative species identifications as well as the habitat type of each site. To control for size, ratios were calculated between shell measurements before use in the ANOVA. These were: height/width, depth1/depth2 and depth 2/width. Tukey's Honestly Significant Difference (HSD) test was used to test statistically between the means of the various groupings, also in R [30].

In order to look for correlation between the various different types of data and even the different types of measurement, distance matrices were constructed and then statistically tested for correlation. For each putative morphological group (‘*B. choanomphala*’, *‘B.choanomphala*-intermediate’, ‘*B. sudanica’* or ‘*B. sudanica*-intermediate’), the mean eigenvalue score for the first two principal components was calculated by averaging the PCA coordinates from all of the individuals within a morphogroup, and was done separately for each morphological analysis. Pair-wise, Euclidean distances between each of the means of the groups was then determined, creating a matrix that could be compared in a pair-wise fashion to the matrix of genetic distance data, using a Mantel r-test.

### Ethical considerations

All necessary permits were obtained for the described field studies from the relevant authorities, namely the Uganda National Council for Science and Technology (UNCST), the Tanzania National Commission for Science and Technology (COSTECH) and the Kenya Ministry of Science and Technology. The field studies did not involve any species for which special permission or clearance was required.

## Supporting Information

Figure S1 **Bayesian inference tree of 16S haplotypes.** Node support (1000 bootstraps or posterior probabilities) from neighbour-joining, Bayesian inference, maximum likelihood and maximum parsimony are given for all nodes which were supported with greater than 50% consensus by any method.
(TIF)

Figure S2 **TCS minimum step-wise distance network of unique 16S haplotypes.** The size of each pie represents the frequency of the haplotype; the colour of the slice symbolises the morphotype of the individuals with that haplotype, as per the key. ‘B_cho morphotype’ and ‘B_sud morphotype’ refer to *B. choanomphala*-like and *B. sudanica*-like snails respectively; ‘B_cho intermed’ and ‘B_sud intermed’ refer to intermediate forms.
(TIF)

Figure S3 **PCA plot showing the first two eigenvalues (‘Factor 1’ and ‘Factor 2’) for the aperture outline analysis.** These two factors account for 61.45% of the variation in the data. The shell pictures demonstrate the visual form of the aperture corresponding to that particular coordinate on the plot. The various different morpho-groups (Lake Victoria samples versus ‘type’ material from Berlin versus field-caught *B. pfeifferi*) are indicated as per the key. The abbreviations for the species names are as follows: ‘B_cho’  =  *B. choanomphala*; ‘B_cho_bas’  =  *B. choanomphala* var. *basinulacatus* ‘B_sud’  =  *B. sudanica*; ‘B_sud_maj’  =  *B. sudanica* var. *major*; ‘B_sud_min  =  *B. sudanica* var. *minor*; ‘intermeds’  =  intermediate forms.
(TIF)

Figure S4 **PCA plot showing the first two eigenvalues (‘Factor 1’ and ‘Factor 2’) for the internal anatomy analysis.** These two factors combined to explain 99.98% of the variation in the data. The various different morpho-groups (in this case, just Lake Victoria samples) are indicated as per the key.
(TIF)

Table S1 (DOCX)
