# Supplementary figures and images for: A Fresh Insight into Transmission of Schistosomiasis: A Misleading Tale of Biomphalaria in Lake Victoria
Source: PLoS One. 2011 Oct 24;6(10):e26563. doi: 10.1371/journal.pone.0026563 (PMC3200340; doi:10.1371/journal.pone.0026563)

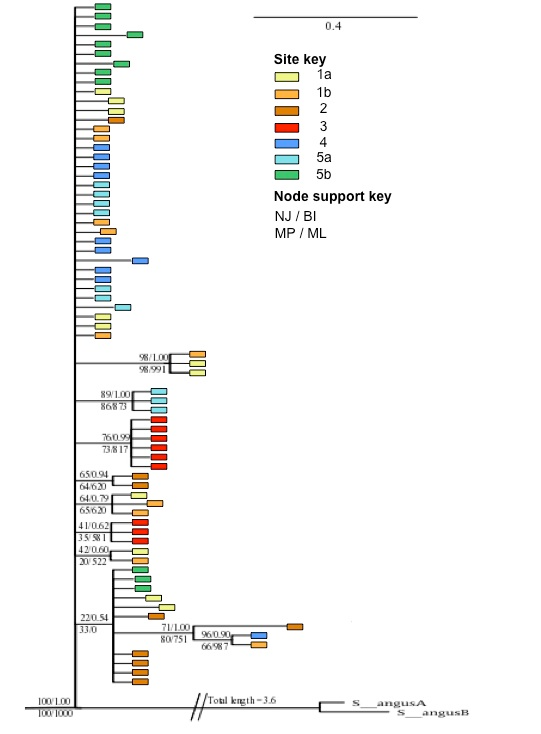

Supplement: Figure S1 — Bayesian inference tree of 16S haplotypes. Node support (1000 bootstraps or posterior probabilities) from neighbour-joining, Bayesian inference, maximum likelihood and maximum parsimony are given for all nodes which were supported with greater than 50% consensus by any method. (TIF) [file pone.0026563.s001.tif]

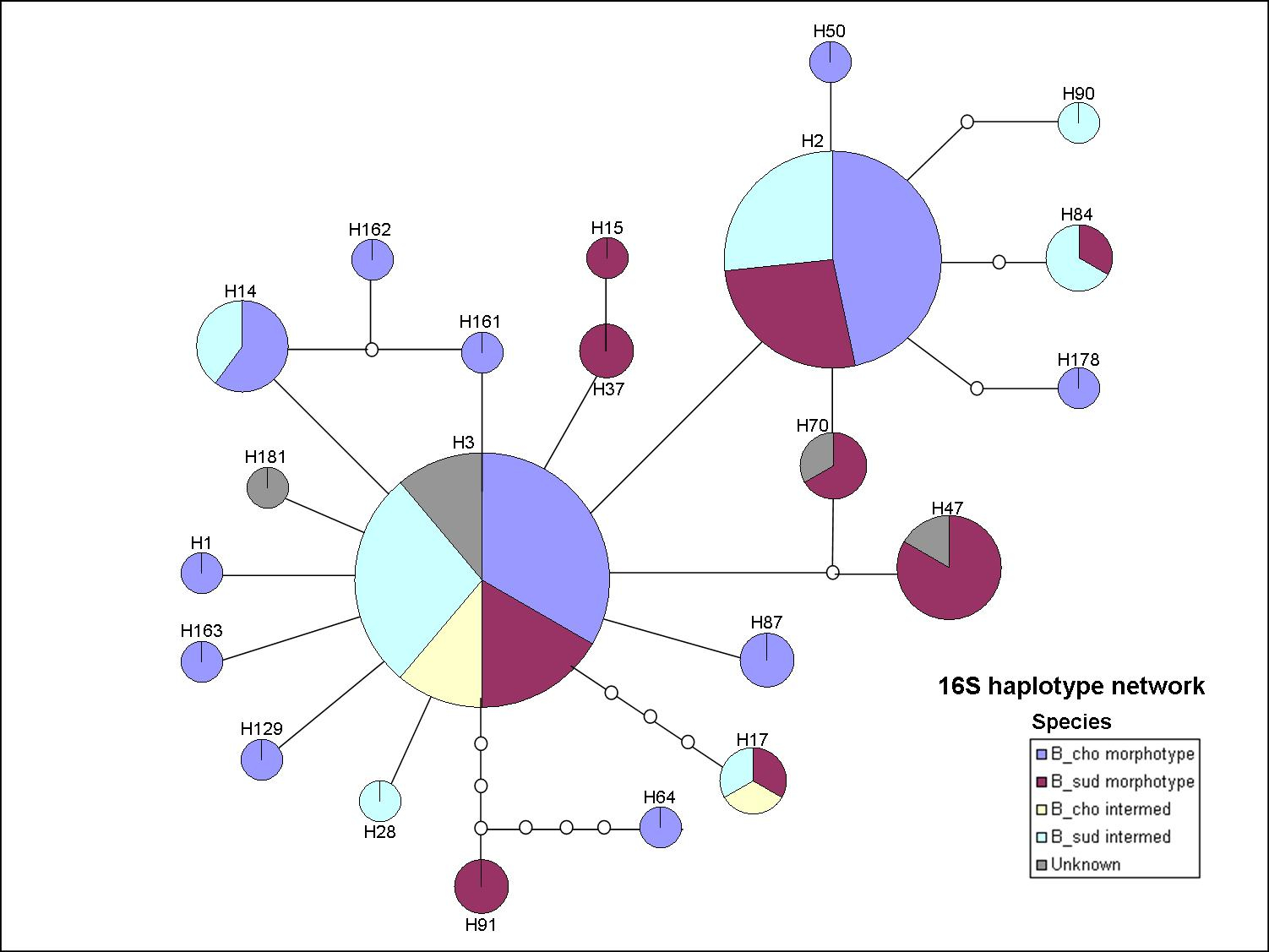

Supplement: Figure S2 — TCS minimum step-wise distance network of unique 16S haplotypes. The size of each pie represents the frequency of the haplotype; the colour of the slice symbolises the morphotype of the individuals with that haplotype, as per the key. ‘B_cho morphotype’ and ‘B_sud morphotype’ refer to B. choanomphala-like and B. sudanica-like snails respectively; ‘B_cho intermed’ and ‘B_sud intermed’ refer to intermediate forms. (TIF) [file pone.0026563.s002.tif]

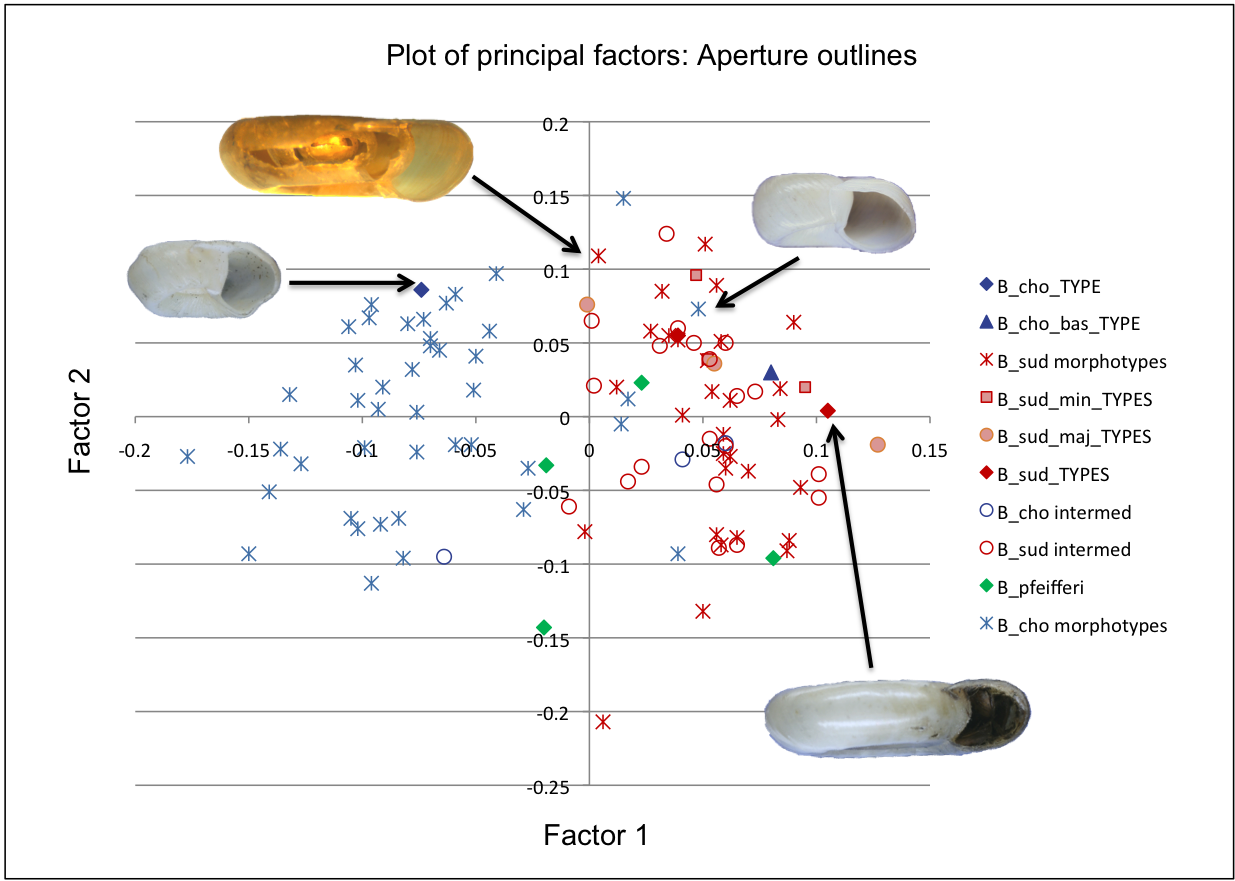

Supplement: Figure S3 — PCA plot showing the first two eigenvalues (‘Factor 1’ and ‘Factor 2’) for the aperture outline analysis. These two factors account for 61.45% of the variation in the data. The shell pictures demonstrate the visual form of the aperture corresponding to that particular coordinate on the plot. The various different morpho-groups (Lake Victoria samples versus ‘type’ material from Berlin versus field-caught B. pfeifferi) are indicated as per the key. The abbreviations for the species names are as follows: ‘B_cho’ = B. choanomphala; ‘B_cho_bas’ = B. choanomphala var. basinulacatus ‘B_sud’ = B. sudanica; ‘B_sud_maj’ = B. sudanica var. major; ‘B_sud_min = B. sudanica var. minor; ‘intermeds’ = intermediate forms. (TIF) [file pone.0026563.s003.tif]

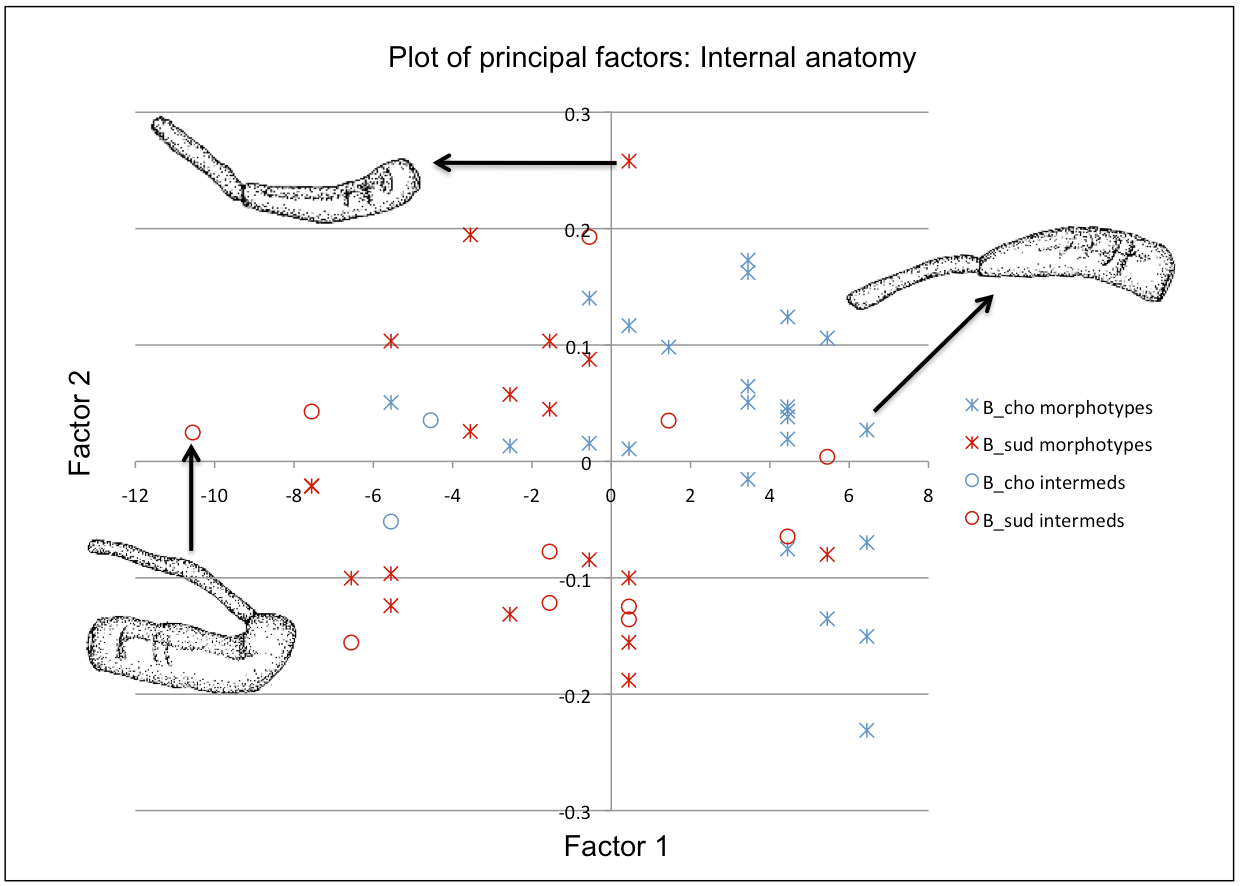

Supplement: Figure S4 — PCA plot showing the first two eigenvalues (‘Factor 1’ and ‘Factor 2’) for the internal anatomy analysis. These two factors combined to explain 99.98% of the variation in the data. The various different morpho-groups (in this case, just Lake Victoria samples) are indicated as per the key. (TIF) [file pone.0026563.s004.tif]
