# Supplementary material for: A Fresh Insight into Transmission of Schistosomiasis: A Misleading Tale of Biomphalaria in Lake Victoria
Source: PLoS One. 2011 Oct 24;6(10):e26563. doi: 10.1371/journal.pone.0026563 (PMC3200340; doi:10.1371/journal.pone.0026563)
Supplement: Table S1 — (DOCX) [file pone.0026563.s005.docx]

| **Morphogroup** | ***B. choanomphala*-like** | ***B. choanomphala*-intermediate** | ***B. sudanica*-like** | ***B. sudanica*-intermediate** |
| --- | --- | --- | --- | --- |
| ***B. choanomphala*-like** | *0.007* |  |  |  |
| ***B. choanomphala*-intermediate** | 0.008 | *0.007* |  |  |
| ***B. sudanica*-like** | 0.008 | 0.008 | *0.008* |  |
| ***B. sudanica*-intermediate** | 0.007 | 0.006 | 0.007 | *0.005* |

**Table S1: 16S ribosomal RNA gene sequence corrected distance measurements within and between morphogroup**

The diagonal (in italics) shows the within-morphogroup mean genetic distance. The model of nucleotide substitution used for the correction was GTR+G.
